# Investigation of Electrical Behavior of Au/Ti/AlN/Si Schottky Diode via Gaussian Distribution Barrier Modeling

**DOI:** 10.1021/acsomega.5c07643

**Published:** 2025-11-07

**Authors:** Abdullah Karaca, Dilber Esra Yıldız, Adem Tataroğlu

**Affiliations:** † Department of Physics, Faculty of Arts and Sciences, 162338 Yozgat Bozok University, 66100 Yozgat, Turkey; ‡ Department of Physics, Faculty of Engineering and Natural Sciences, 583633 Hitit University, 19030 Çorum, Turkey; § Department of Chemistry, Faculty of Arts and Sciences, Middle East Technical University, 06800 Ankara, Turkey; ∥ Department of Physics, Faculty of Science, 37511 Gazi University, 06500 Ankara, Turkey

## Abstract

This study presents a comprehensive analysis of the temperature-dependent electrical behavior of Au/Ti/AlN/n-Si Schottky diode using both experimental measurements and theoretical modeling. The key diode parameters, namely the ideality factor (*n*) and the zero-bias barrier height (ϕ_*B*0_) were extracted over the temperature range of 100–400 K and were found to exhibit a strong dependence on temperature. The experimental results revealed that “ϕ_*B*0_” increases and “*n*” decreases with rising temperature, consistent with the presence of a Gaussian distribution of barrier heights and a transition toward thermionic emission-dominated transport. A theoretical model incorporating barrier inhomogeneities was employed and yielding results in close agreement with experimental trends, particularly for ϕ_*B*0_. Modified Richardson plots confirmed the validity of the Gaussian distribution model, allowing for accurate determination of the average barrier height and Richardson constant. While the theoretical predictions matched the experimental behavior well at high temperatures, deviations at lower temperatures showed the influence of interface states and nonideal effects. The observed behavior indicates that the charge transport mechanism is influenced by spatial variations in the potential barrier, particularly at lower temperatures.

## Graphical abstract

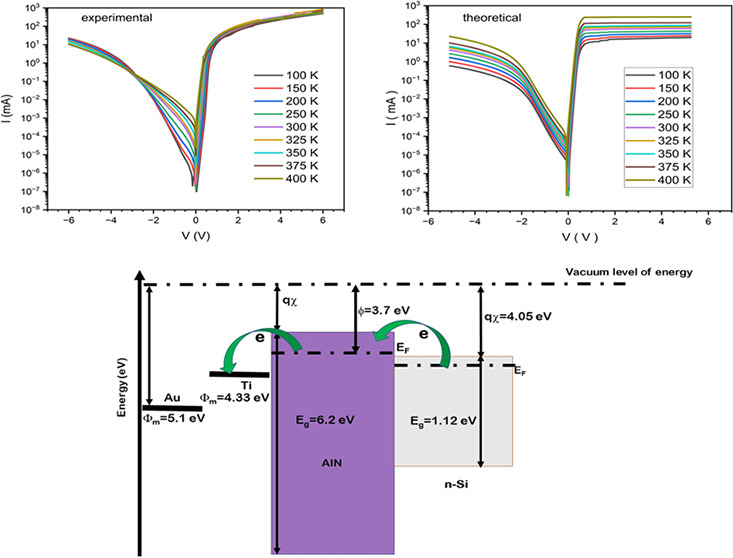

## 1 Introduction

Metal–semiconductor (MS) junctions play a fundamental role in modern electronics and optoelectronic technologies, forming the fundamental building blocks of devices such as Schottky diodes, photodetectors, and high-speed rectifiers. Their widespread application stems from their relatively simple structure, fast switching capabilities, and compatibility with a variety of semiconductor platforms.^1,2^ Despite their structural simplicity, MS junctions often exhibit complex electrical behavior under nonideal conditions, particularly those affected by interface phenomena, temperature variations, and material defects.^3,4^ These deviations from ideality highlight the importance of a detailed understanding of carrier transport mechanisms, barrier formation, and interface quality in MS-based device structures. Despite the apparent simplicity of the MS structure, deviations from ideal diode behavior are frequently observed due to various interface-related phenomena. These include interface states, oxide layers, and spatial inhomogeneities in the Schottky barrier height (SBH), which critically affect carrier transport mechanisms particularly under varying temperature conditions. Such inhomogeneities are often attributed to structural imperfections and local variations in the interfacial potential, which necessitate advanced models beyond the conventional thermionic emission theory. One of the most effective approaches to describe this nonideal behavior is the Gaussian distribution (GD) model, which accounts for the presence of multiple barrier heights at the interface.^5–8^

A deeper understanding of the electrical response of metal–semiconductor (MS) diodes requires particular attention to fundamental diode parameters, most notably the ideality factor (*n*) and the zero-bias barrier height (φ). The ideality factor is widely recognized as a diagnostic parameter that quantifies the degree of deviation from the ideal thermionic emission model.^9,10^ Values of n greater than unity generally indicate the involvement of additional carrier transport mechanisms such as interface recombination, tunneling through localized states, image-force lowering, or spatial inhomogeneities at the MS interface. Thus, a systematic evaluation of n provides critical informations into the nature and extent of nonidealities that govern charge transport. Similarly, the zero-bias barrier height (ϕ) constitutes a fundamental descriptor of the Schottky contact quality, directly influencing the magnitude of the forward current, the rectification ratio, and ultimately the efficiency of device operation.^11^ Variations in φ with temperature or bias are frequently correlated with barrier inhomogeneities, interfacial defects, or dipole layers, which in turn reflect the microscopic quality of the interface. Accordingly, careful analysis of both *n* and ϕ under temperature-dependent conditions not only enables the identification of dominant conduction mechanisms but also serves as a robust framework for assessing the reliability, reproducibility, and potential applicability of MS-based devices in practical electronic and optoelectronic systems.

Aluminum nitride (AlN) has garnered significant attention in recent years due to its exceptional material properties, making it an attractive candidate for advanced electronic and optoelectronic device applications.^12–14^ As a wide-bandgap semiconductor (∼6.2 eV), AlN exhibits high thermal conductivity, excellent electrical insulation and outstanding chemical stability under extreme environmental conditions. These characteristics render it particularly suitable for high-frequency, high-temperature, and high-power electronic devices. Moreover, AlN films grown with high crystalline quality serve as efficient buffer or interfacial layers in metal–semiconductor (MS) structures, where they contribute to interface passivation, reduced trap densities, and enhanced carrier transport.^15–17^ The intrinsic wide bandgap and high breakdown field also enable AlN to sustain large electric fields without premature failure, which is critical for reliable device performance. Due to its compatibility with various deposition techniques and substrates,^18,19^ AlN-based heterostructures offer great potential for tailoring interface energetics, thereby allowing precise control over Schottky barrier formation and tuning of device characteristics.^20–22^ Recent studies have demonstrated progress the potential of AlN-based nanostructures and devices for photodetection.^23,24^ For instance, the absolute responsivity of a metal–semiconductor–metal (MSM) photodiode fabricated from high-quality AlN was evaluated across the VUV to near-UV range (44–360 nm), where the device, employing Schottky metal finger contacts with 2 μm width and 4 μm spacing, exhibited excellent sensitivity, stability, and a 200/360 nm rejection ratio exceeding 4 orders of magnitude, underscoring the advantages of UWBG semiconductors for solar observation missions.^25^ Complementary to this, Zheng et al. reported the synthesis of defect-free and highly crystalline AlN micro/nanowires through a two-step physical vapor transport (PVT) method that combined high-temperature growth with an auxiliary annealing process to effectively eliminate point defects. The as-prepared AlN nanostructures, confirmed by structural analyses, were subsequently employed to fabricate a low-dimensional VUV-sensitive photodetector with an ultrashort cutoff wavelength of 208 nm and ultrafast response speeds (<0.1 s rise and <0.2 s recovery), outperforming thin-film AlN devices by 1–2 orders of magnitude.^26^ Together, these findings highlight the significant progress toward compact, stable, and high-performance AlN-based VUV detectors, paving the way for cost-effective and energy-efficient sensing technologies in space and solar applications. In this context, the incorporation of AlN as an interlayer within MS configurations presents a promising pathway to engineer rectifying behavior and thermal response, particularly under varying environmental and operational conditions.

This study presents a comprehensive investigation of the temperature-dependent current–voltage (*I*–*V*) characteristics of a diode structure based on Si, incorporating an AlN thin film as an interfacial layer. The effects of barrier inhomogeneities, are evaluated in detail to elucidate their influence on the current transport behavior via modeled GD. The charge carrier conduction mechanism and potential barrier profile at the metal–semiconductor (MS) junction were systematically examined over a wide temperature range between 100 and 400 K. Despite the structurally simple MS configuration, the electrical response of the diode deviates from ideal behavior due to the influence of series resistance and the presence of interface trap states. Variations in interface quality and material properties across the layers were found to induce local fluctuations in barrier height, thereby leading to nonideal diode behavior characterized by ideality factors exceeding unity. The diode structure was engineered by employing a rectifying Ti/Au contact on an AlN-based diode structure. The device was subjected to temperature-dependent electrical measurements to assess both its performance parameters and thermal response sensitivity. While existing literature on MS-based structures largely focuses on the interface characteristics and barrier analysis, this work offers a distinctive contribution by addressing the anomalous carrier transport behavior within the scope of a Gaussian-distributed barrier model with experimental and theoretical approach. The existence and spatial distribution of localized low-barrier regions around the mean barrier height were shown to significantly impact the dominant conduction pathway. Experimentally determined values of barrier height and ideality factor were compared with theoretical estimations. The findings clearly underscore the crucial role of barrier inhomogeneities in defining the electrical performance of the diode, offering insights into the nature of temperature-dependent charge transport mechanisms in MS-based devices.

## 2 Numerical and Experimental Methods

The AlN/n-Si heterostructure used in this study was commercially sourced from MTI Corporation and employed directly in Schottky diode fabrication following a cleaning protocol. The n-type silicon (Si) substrate exhibited a (111) orientation, with a diameter of 2 in., a thickness of 0.5 mm, and a resistivity in the range of 1–10 Ω·cm. The AlN thin film, 200 nm in thickness, was deposited on the Si wafer via hydride vapor phase epitaxy (HVPE). Prior to device fabrication, the wafer was pieced into 1 × 2 cm^2^ pieces and sequentially cleaned in an ultrasonic bath using 2-propanol, acetone, and deionized water. The cleaned substrates were dried under a nitrogen stream and subjected to plasma treatment for 5 min to enhance surface cleanliness and activation. To establish an ohmic back contact, a 100 nm thick gold film was deposited onto the rear side of the n-Si via thermal evaporation under a high vacuum (∼10^–6^ Torr), followed by rapid thermal annealing at 500 °C for 5 min in a nitrogen ambient. On the AlN surface, circular Schottky contacts were formed using a patterned hole-array mask, through which a bilayer of Ti (5 nm) and Au (100 nm) was deposited via thermal evaporation. The use of the Ti/Au stack was selected to reduce the Schottky barrier height, leveraging the intermediate work function of the bilayer compared to individual Au or Ti contacts, as previously reported.^27–31^ This configuration resulted in a vertically aligned Au/Ti/AlN/n-Si heterojunction structure suitable for electronic analysis. In our previous work, Kocyigit et al.^30^ reported analyses of identical AlN/n-Si heterostructures, demonstrating a uniform surface morphology with nanoscale porosity. Electrical characterization was conducted at the temperature range of 100–400 K conditions using a Keithley 2400 source-meter, Lake Shore model 321 autotuning temperature controller and VPF-475 cryostat.

The threshold voltage (*V*_th_), defined as the forward bias voltage at which current conduction sharply increases, was extracted from the *I*–*V* characteristics and found to range between 0.2 and 0.3 V, which is consistent with typical values for Schottky-type diodes. In order to evaluate the response performance of the diode, current measurements were systematically recorded under varying temperatures.

To gain a deeper understanding of the optoelectronic behavior of the proposed device structure, numerical simulations were conducted using the SCAPS-1D (Solar Cell Capacitance Simulator) software.^32^ SCAPS-1D is a well-established one-dimensional simulator extensively utilized for analyzing the optical and electrical characteristics of multilayered semiconductor devices.^33,34^ In this study, simulations were performed under illumination using a fixed spectrum source file (JL.spe), which approximates the standard AM1.5G solar spectrum. According to the literature, results obtained from SCAPS-1D simulations exhibit strong agreement with experimental findings, thereby validating the reliability of this computational tool for device optimization. The simulator solves a coupled system of three fundamental differential equations self-consistently using an iterative method: Poisson’s equation (eq 1),^35^ and the continuity equations for electrons (eq 2)^36^ and holes (eq 3),^37^ subject to appropriate boundary conditions. These equations are expressed as follows

1 $$\frac{\partial^{2}\Psi }{\partial x^{2}}+\frac{q}{\varepsilon }\left\{p\left(x\right)-n\left(x\right)+N_{D}\left(x\right)-N_{A}\left(x\right)+p_{t}\left(x\right)-n_{t}\left(x\right)\right\}=0$$

2 $$\frac{dJ_{p}}{dx}=G_{p}\left(x\right)-R\left(x\right)$$

3 $$\frac{dJ_{n}}{dx}=-G_{n}\left(x\right)+R\left(x\right)$$

In the context of semiconductor device modeling; ε represents the dielectric permittivity, Ψ is the local electric potential and *q* denotes the elementary charge. the spatially dependent functions *p*(*x*) and *n*(*x*) represent the concentrations of free holes and electrons, respectively. The terms *N*_*D*_(*x*) and *N*_*A*_(*x*) correspond to the distributions of ionized donor and acceptor impurities. Additionally, *p_t_* (*x*) and *n_t_* (*x*) denote the localized trap state densities associated with holes and electrons, which influence carrier dynamics through trapping and detrapping processes. The quantities *J*_*n*_ and *J*_*p*_ describe the electron and hole current densities, while *G*_*n*/*p*_ and *R*_*n*/*p*_ account for position-dependent carrier generation and recombination mechanisms, playing a central role in the charge transport and continuity equations.

The fabricated Au/Ti/AlN/Si Schottky diode and the energy band diagram were presented in Figure 1a,b, respectively. The energy alignment shows the barrier height at the Au/Ti/AlN/Si interface, which governs charge carrier transport and determines key diode parameters such as the turn-on voltage and rectification behavior. A comprehensive understanding of the band structure is essential for accurately interpreting the temperature-dependent charge transport mechanisms and the associated electrical behavior of the Schottky diode, as elaborated in the following sections. Table 1 presents the values of various physical parameters employed in the different layers of the AlN-based diode, as extracted from previously published literature sources. This study aims to systematically investigate the influence of critical parameters on the performance of AlN layer. To this end, key device parameters including the thickness of the absorber layer, the doping concentration, and the total defect density within the AlN layer were varied, and their effects on the optoelectronic performance were thoroughly examined through numerical simulation. Following a comprehensive parametric analysis, the optimal values for these parameters were determined. Based on these optimized conditions, AlN diodes were subsequently fabricated, and their performance metrics were experimentally characterized. Furthermore, the temperature-dependent optimization conditions were evaluated by conducting comparative current–voltage (*I–V*) measurements, enabling a deeper understanding of the thermal behavior of the devices.

**Figure 1:**
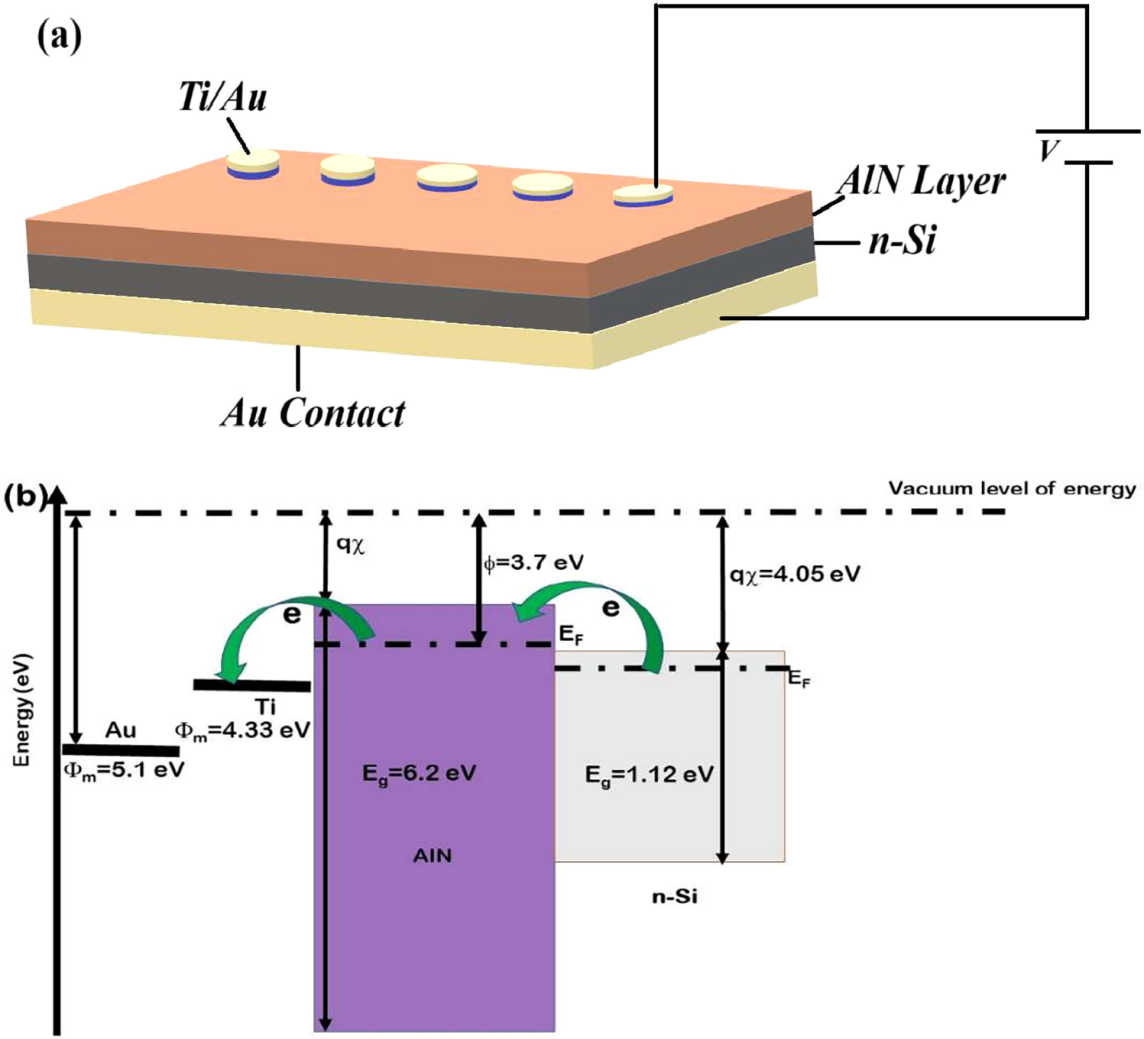
(a) Schematic diagram of the Au/Ti/AlN/Si Schottky diode structure; (b) corresponding energy band diagram illustrating the device.

**Table 1:** Input Parameters Defined for Layers in SCAPS-1D Simulation Environment^19,38–40^

devices parameters	n-type Si	AlN
layer thickness (μm)	300	variable
band gap (eV)	1.12	6.2
electron affinity, χ (eV)	4.05	3.7
relative permittivity, (ε_r_)	11.9	9
effective DOS in CB, *N*_c_ (cm^–3^)	2.8 × 10^19^	3.1 × 10^18^
effective DOS in VB, *N*_v_ (cm^–3^)	1.04 × 10^19^	1.8 × 10^19^
electron mobility, μ_n_ (cm^2^/(V s))	1350	100
hole mobility, μ_p_ (cm^2^/(V s))	500	30
energetic distribution	Gaussian	Gaussian
electron and hole thermal velocity (cm/s)	10^7^	10^7^
doping concentration density (cm^–3^)	7.5 × 10^15^	1 × 10^17^
electron surface recombination velocity (cm/s)	10^7^	10^5^
operating temperature range (K)	100–400	

## 3 Results and Discussion

The current–voltage (*I*–*V*) characteristics of a device provide insight into its charge transport behavior under externally applied bias. For Schottky-type devices and conventional diodes, current conduction typically occurs beyond a threshold voltage in the forward bias region, while it remains negligible in reverse bias under dark conditions.^41,42^ Figure 2a,b. respectively present the experimental and theoretical *I*–*V* characteristics of the Au/Ti/AlN/n-Si heterostructure under varying temperatures. Both data sets reveal a noticeable increase in the forward and reverse current with rising temperature, indicating that the heterostructure exhibits a thermally activated charge transport mechanism.^43^ This suggests that the carrier concentration increases with thermal excitation. In the experimental data shown in Figure 2a., a clear logarithmic increase in current is observed in both forward and reverse biases as the temperature rises from 100 to 400 K. Particularly in the reverse direction, the current remains significantly low at low temperatures but increases markedly at higher temperatures. This behavior can be attributed to the limited thermionic emission at low temperatures due to insufficient thermal energy of carriers.^44^ Moreover, the spread in the reverse-bias current observed in the experimental curves indicates the influence of interface defects and trap states. In contrast, the theoretical curves in Figure 2b. display a smoother and more uniform increase in current with temperature. This regularity suggests that the theoretical model excludes real-devise parameters such as interface traps, series resistance effects, and structural imperfections. Especially in the reverse-bias region, the theoretical curves are more defined and stable, whereas the experimental curves show greater deviations and irregularities.^45^ These discrepancies may result from physical factors such as structural inhomogeneity in the device, potential barriers introduced by the AlN interlayer, and diffusion-related effects.^27^ Additionally, both experimental and theoretical plots demonstrate a sharp increase in current beyond a certain threshold voltage (*V*_th_) in the forward direction. However, this transition appears sharper and more abrupt in the theoretical model, while in the experimental data, the increase is more gradual and diffused. This implies that additional phenomena such as diffusion current, surface effects, and contact resistance are at play in the real system. The experimental data reveals a distinct crossover point in the reverse bias region near −3 V. This feature, which is conspicuously absent from the theoretical model, is a well-established signature of a transition in the dominant carrier transport mechanism. At lower reverse bias, the current is primarily governed by thermally activated processes like thermionic emission and generation-recombination. However, as the reverse bias increases, the strong electric field activates field-driven mechanisms, including trap-assisted tunneling and Poole-Frenkel emission.^46^ These latter processes exhibit a weaker, or even inverse, dependence on temperature due to trap depopulation and enhanced carrier escape from localized states. This competition between thermally driven and field-driven pathways leads to the convergence and eventual intersection of the *I*–*V* curves, a hallmark of inhomogeneous interfaces with significant defect-mediated conduction under high-field conditions. The presence of this crossover provides critical experimental evidence for the increasing dominance of nonideal, field-enhanced transport mechanisms in the high reverse-bias regime, reflecting the complex interplay between temperature, electric field, and defect states at the metal–interlayer–semiconductor interface. Although the general trends of the experimental and theoretical *I–V* characteristics are consistent with each other, the experimental results reflect the real physical and structural influences on the devices particularly the temperature-dependent defects at the heterointerface.^47^ These differences are crucial for understanding the complex transport processes encountered under real operating conditions of the AlN-based heterostructures.

**Figure 2:**
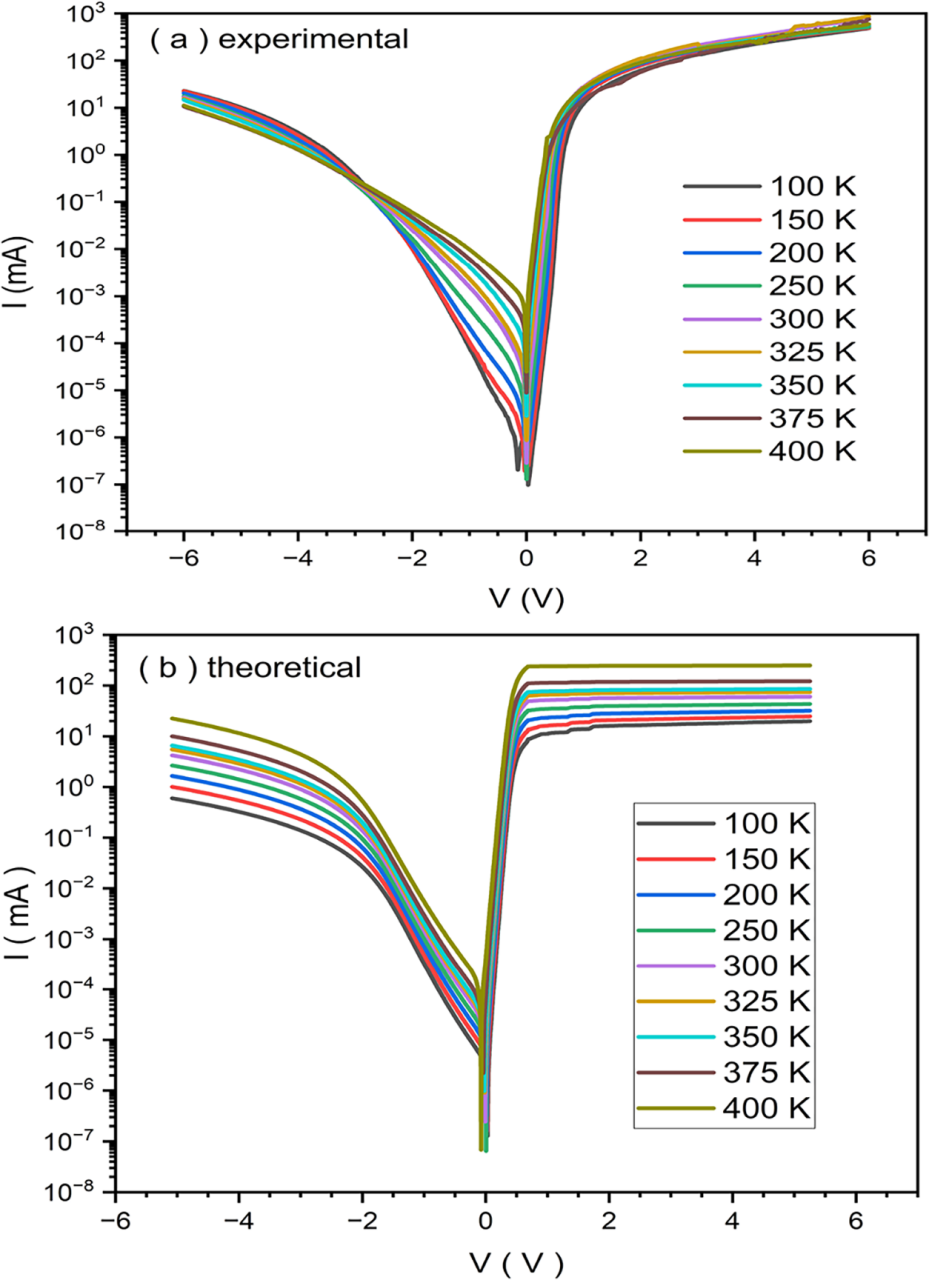
(a) Experimental, (b) theoretical *I–V* characteristics at various temperatures.

The current–voltage (*I*–*V*) characteristics of the AlN/n-Si heterostructure diode were initially analyzed using the ideal thermionic emission (TE) model, which assumes a uniform barrier height and negligible recombination or tunneling effects.^48^ According to this model, the diode current is expressed by the eq 4.

4 $$I=I_{0}\left(\exp\left(\frac{\mathrm{qV}}{\mathrm{nkT}}\right)-1\right)$$

Here, *I* denotes the forward bias current, *I*_0_ is the reverse saturation current, *q* represents the elementary charge, V is the applied voltage, *n* is the ideality factor, *k* is Boltzmann’s constant, and *T* corresponds to the absolute temperature. The ideality factor (*n*) provides insight into the deviation of the diode from pure thermionic behavior, with *n* = 1 representing an ideal diode. However, deviations from this ideal model for Schottky contacts (mostly due to interface states, image strength reduction, or spatial barrier inhomogeneities) require more advanced modeling approaches such as Gaussian barrier dispersion models.^49,50^ In the presence of interfacial layers, the current transport mechanism in the Schottky junction can still be primarily described by the thermionic emission model under forward bias conditions. According to eq 4, the reverse saturation current (*I*_0_) is a critical parameter that reflects the barrier properties at zero bias. Experimentally, the reverse saturation current (*I*_0_) is determined by extrapolating the linear portion of the forward bias ln­(*I*)–*V* curve to the origin (*V* = 0). This approach enables an accurate estimation of *I*_0_, which for evaluating the effective barrier height (ϕ_*B*0_) and for gaining insight into the charge transport mechanisms across the metal–semiconductor interface.^51^ The incorporation of ultrathin dielectric interlayers can alter the observed barrier characteristics by influencing interfacial states, modifying the tunneling probability, and introducing polarization effects. These factors collectively contribute to the deviation in *I*_0_ values obtained from experimental observations.^52–56^

5 $$I=AA^{*}\exp\left(-\frac{e\phi_{B0}}{\mathrm{kT}}\right)\left(\exp\left(\frac{\mathrm{eV}}{\mathrm{nkT}}\right)-1\right)$$

6 $$n=\frac{q}{\mathrm{kT}}\left(\frac{dV}{d\ln I}\right)$$

7 $$\phi_{B0}=\frac{\mathrm{kT}}{q}\left(\frac{AA^{*}T^{2}}{I_{0}}\right)$$

8 $$A^{*}=4\pi m^{*}k^{2}/h^{3}=120\left(m^{*}/m_{0}\right)$$

Within this framework, the current density observed under forward bias in Schottky diodes depends primarily on three parameters: the effective diode area (*A*), the Richardson constant (A*), and the Schottky barrier height at zero bias (ϕ_*B*0_). The Richardson constant is derived based on the nearly free electron approximation, assuming vacuum-like conditions, and is expressed as eq 8. where *h* is Planck’s constant, *k* is Boltzmann’s constant, *q* is the elementary charge, *m*_0_ is the free electron mass, and m* is the effective mass of the majority carrier in the semiconductor. This parameter directly reflects the specific material properties of the semiconductor. Additionally, the ideality factor (*n*) quantitatively indicates the deviation of the actual diode from ideal thermionic emission behavior. Such deviations are often attributed to physical mechanisms like interface states, tunneling effects, or spatial fluctuations in the barrier height. Therefore, the analysis of the “*n*” provides essential insight into the interface quality and underlying transport phenomena.^57–60^

Figure 3a,b. present the temperature-dependent variations of the ideality factor (*n*) and zero-bias barrier height (ϕ_*B*0_) of Au/Ti/AlN/n-Si Schottky diodes, obtained experimentally and theoretically, respectively. In both plots, it is clearly observed that the ideality factor decreases significantly with increasing temperature, while the zero-bias barrier height increases accordingly. This behavior is attributed to barrier height inhomogeneities at the metal–semiconductor interface, which become more prominent with increasing temperature.^6,43^ Experimentally, the ideality factor decreases from approximately 4.8 at low temperatures to around 1.2 at 400 K, whereas the ϕ_*B*0_ value increases from roughly 0.27 to 0.88 eV. This trend suggests that the Schottky barrier height is not uniform but rather follows a Gaussian distribution. As the temperature rises, charge carriers are more likely to overcome higher barrier regions, resulting in increased ϕ_*B*0_ and decreased *n* values. At low temperatures, carriers tend to traverse lower barrier regions, leading to nonideal behavior (*n* > 1), whereas at higher temperatures, carrier transport shifts toward more homogeneous regions, approaching ideal diode characteristics (*n* ≈ 1).^61,62^ The theoretical modeling shown in Figure 3b. demonstrates a similar trend. The ideality factor starts at about 3.2 and decreases to 1.0 at 400 K, reflecting a more ideal interface assumed under theoretical boundary conditions. Additionally, the theoretical ϕ_*B*0_ values are close to the experimental ones but begin at a slightly higher point and exhibit a more linear increase with temperature. Overall, both graphs reveal that the electrical properties of the Au/AlN/n-Si heterostructure are highly sensitive to temperature and that the carrier transport mechanism is strongly influenced by the spatial distribution of the barrier height at the interface. The strong agreement between experimental and theoretical data supports the physical validity of the applied model. Such temperature-dependent investigations provide deeper insight into Schottky contact behavior and demonstrate the potential suitability of these heterostructures for optoelectronic device applications.^63^ At lower temperatures, particularly around 100 K, the ideality factor reaches notably high values, with the experimental data approaching *n* ≈ 4.9, while the theoretical value is slightly lower, at approximately 3.3. Such elevated values at low temperatures suggest significant deviation from ideal Schottky behavior, which is typically attributed to barrier height inhomogeneities at the metal–semiconductor junction. In this regime, carriers are more likely to traverse regions of lower barrier height, leading to an increase in the apparent ideality factor. As the temperature increases, the ideality factor progressively decreases, eventually approaching unity at higher temperatures (above 350 K). This behavior implies that thermionic emission becomes the dominant transport mechanism, and carriers acquire sufficient thermal energy to surmount higher potential barriers.^64,65^ Consequently, the impact of spatial barrier fluctuations diminishes, and the diode exhibits a more ideal Schottky response. The close agreement between experimental and theoretical data further confirms the reliability of the modeling approach employed and supports the interpretation that the temperature-dependent behavior of the ″*n″* arises from the interplay between thermionic emission and barrier inhomogeneity effects.

9 $$P\left(\phi_{B0}\right)=\frac{1}{\sigma \sqrt{2\pi }}\exp\left(-\frac{{\left(\phi_{B0}-\overset{®}{\phi_{B0}\;}\right)}^{2}}{{2\sigma }^{2}}\right)$$

Equation 9 defines the Gaussian probability density function (GDF) that models the spatial distribution of Schottky barrier heights (SBHs) across the metal–semiconductor interface.^7^ Here, *P*(ϕ_*B*0_) represents the probability that a local barrier height takes the value ϕ_*B*0_, while ϕ_*B*0_ and σ correspond to the mean barrier height and its standard deviation, respectively. This statistical treatment captures the intrinsic inhomogeneity of the interface, where microscopic fluctuations and imperfections generate a range of barrier heights rather than a single uniform value. The Gaussian distribution is justified by its capacity to approximate random variations around the mean, reflecting physical realities such as interface roughness, compositional variations, and defect-induced potential fluctuations.^66–68^ The effective barrier height governing charge transport is not a fixed quantity but rather a weighted average over this distribution, which profoundly influences the temperature and voltage dependence of the diode’s electrical characteristics. According to this model, an inhomogeneous Schottky barrier height characterized by an abnormal increase in ϕ_*B*0_ and a decrease in the ideality factor *n* with increasing temperature can be described using a Gaussian-type distribution. This approach assumes the presence of spatially distributed patches with varying barrier heights, which interact and contribute collectively to the total current. Figure 4a,b. presents a comparative analysis of the ideality factor (*n*) as a function of (a) temperature and (b) the inverse temperature (1000/*T*), based on both experimental and theoretical data. The graph reveals a clear trend: as the temperature decreases (i.e., as 1000/*T* increases), the “*n*” increases significantly. This behavior is consistent with thermally activated transport mechanisms, where reduced thermal energy leads to a higher degree of carrier localization and accumulation. A close agreement between the experimental and theoretical data is evident across most of the examined temperature range, particularly between 1000/*T* ≈ 4 and 7. In this interval, the theoretical model appears to accurately capture the dominant physical processes governing carrier dynamics. The minor deviations observed at higher values of 1000/*T* (corresponding to lower temperatures) may be attributed to secondary effects not fully accounted for in the theoretical formulation such as trap-assisted conduction, surface states, or quantum confinement phenomena.^69,70^

**Figure 3:**
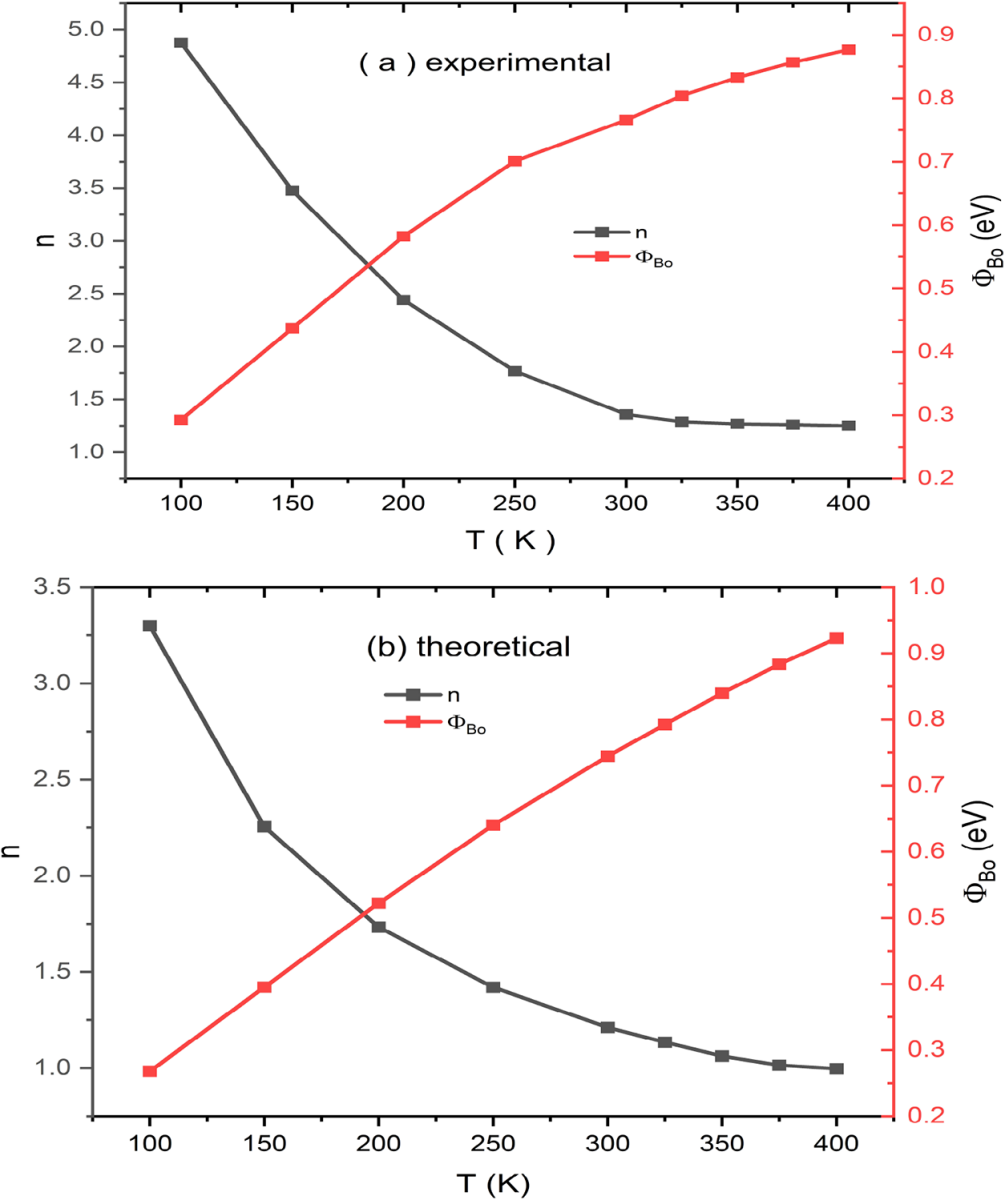
(a) Experimental (b) theoretical temperature-dependent ideality factor (n) and zero-bias barrier height (ϕ_*B*0_) of AlN/n-Si diodes.

**Figure 4:**
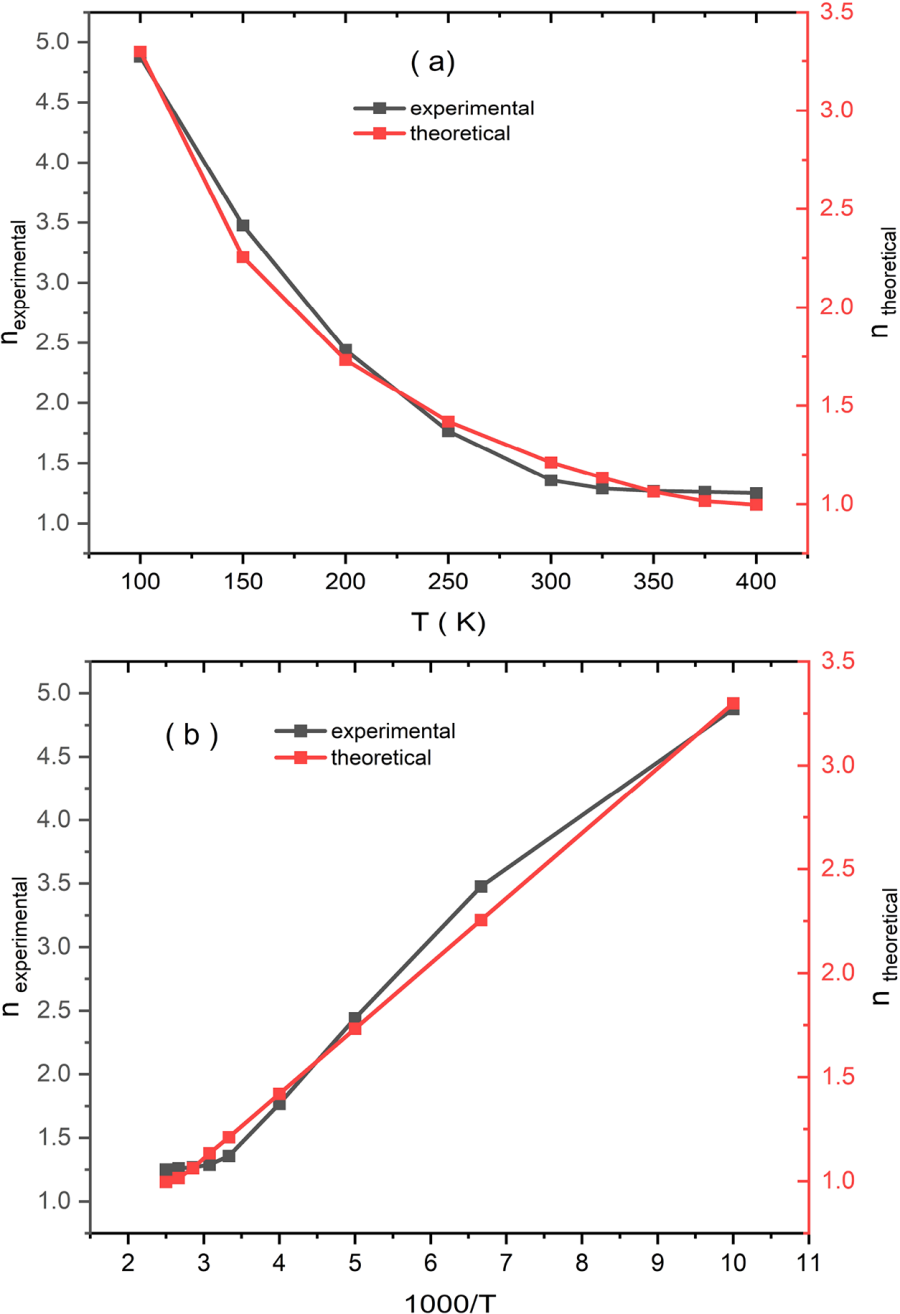
Comparative analysis of the ideality factor (*n*) as a function of (a) temperature (*T*) and (b) inverse temperature (1000/*T*).

Under the Gaussian distribution (GD) approximation of the Schottky barrier height inhomogeneity, the conventional Richardson equation derived from the pure thermionic emission (TE) model requires modification. In ideal cases, the Richardson constant A* is typically extracted by plotting ln (*I*_0_/*T* ^2^) versus the reciprocal temperature (1/*T*), yielding a linear relationship according to eq 2. However, when accounting for barrier inhomogeneity effects, this classical approach must be revisited. By incorporating the modified current–voltage relations expressed in eqs 10 and 11, which include the Gaussian distribution parameters,^71^ the Richardson plot is adjusted accordingly. Consequently, the modified Richardson plot retains linearity but reflects the statistical nature of the barrier height fluctuations, enabling more accurate extraction of both the effective Richardson constant and the mean barrier height for nonideal Schottky contacts. Figure 5a,b. present Arrhenius-type plots based on the thermionic emission model, comparing the experimental and theoretical values of the saturation current (*I*_0_), respectively.^72^ Both graphs depict the natural logarithm of *I*_0_/*T* ^2^ as a function of *q*/*kT*, where *q* is the elementary charge, *k* is the Boltzmann constant, and *T* is the absolute temperature. Linear fitting in the high-temperature region enables the extraction of key diode parameters such as the activation energy (*E*_a_) and the effective modified Richardson constant (A*). In Figure 5a., the experimental data yield a linear region with the relation

10 $$I_{0}=AA^{*}\exp\left(-\frac{q\phi_{\mathrm{app}}}{\mathrm{kT}}\right)$$

11 $$\phi_{\mathrm{app}}=\phi_{B0\left(\mathrm{average}\right)}\left(T=0\right)-\frac{q\sigma^{2}}{2\mathrm{kT}}$$

12 $$\ln\left(I_{0}/T^{2}\right)=\left(-0.3987\right)q/\mathrm{kT}-14.305$$

from which an activation energy of *E*_a_ = 0.399 eV and a modified Richardson constant of A* = 7.81 × 10^–5^ A/cm^–2^K^–2^ are derived. The relatively high activation energy suggests a dominant thermally activated conduction mechanism at elevated temperatures. Moreover, the magnitude of the modified Richardson constant is notably lower than the ideal theoretical value for conventional semiconductors, which may indicate the presence of interfacial barriers, surface states, or structural defects influencing the carrier transport.^73^ In contrast, Figure 5b. shows the theoretical data, described by the relation

13 $$\ln\left(I_{0}/T^{2}\right)=\left(-0.1651\right)q/\mathrm{kT}-22.35$$

corresponding to a significantly lower activation energy of *E*_a_ = 0.165 eV and a modified Richardson constant of A* = 2.5 × 10^–8^ A/cm^–2^K^–2^. The substantially lower activation energy in the theoretical model implies a less temperature-sensitive carrier emission process, possibly due to oversimplified assumptions in the theoretical framework or the exclusion of critical temperature-dependent scattering mechanisms.^74^ Furthermore, the order-of-magnitude difference in A* between theory and experiment shows a clear deviation, likely arising from idealized boundary conditions, geometric factors, or material inhomogeneities not captured in the model.^75,76^ While both plots exhibit a linear behavior in the high-temperature regime—validating the use of the thermionic emission model—quantitative discrepancies between experimental and theoretical results highlight the importance of incorporating real physical effects into theoretical modeling. These include but are not limited to interface recombination, barrier inhomogeneities, and trap-assisted conduction mechanisms, all of which can significantly impact the accuracy of parameter extraction and device performance prediction.

**Figure 5:**
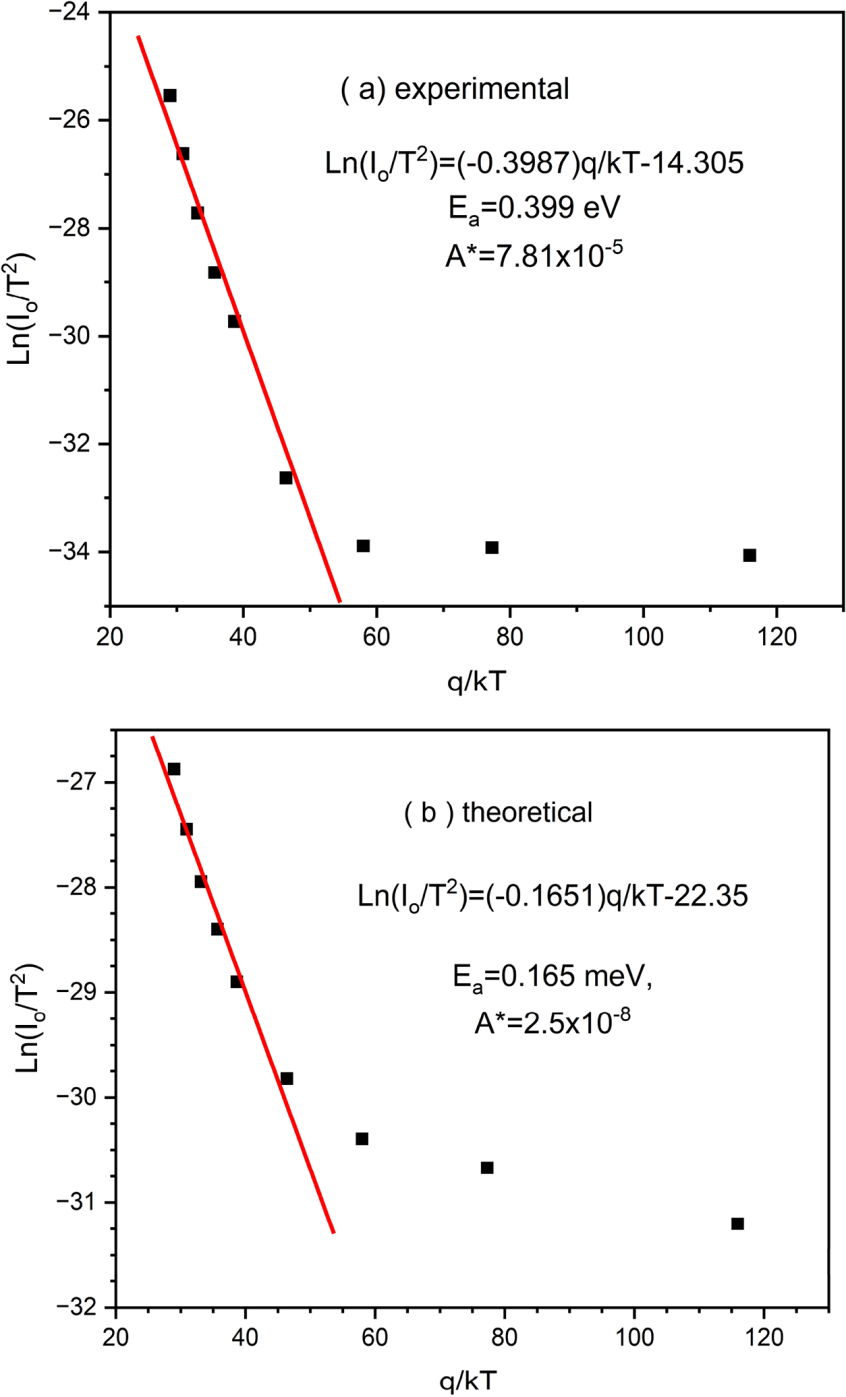
Comparative analysis of the (a) experimental (b) theoretical ln­(*I*_0_/*T* ^2^) versus q/*kT* AlN/n-Si Schottky diode.

Figure 6a,b present the variation of the “ϕ_*B*0_” as a function of the inverse thermal energy (*q*/2*kT*) for experimental and theoretical data sets, respectively. These plots are typically employed to analyze barrier inhomogeneities within the framework of the Gaussian distribution model. In Figure 6a, the experimental data reveal a linear dependence of ϕ_*B*0_ on *q*/2*kT*, yielding the following empirical relation

14 $$\phi_{B0}=\left(-0.0131\right)q/2\mathrm{kT}+1.0442$$

**Figure 6:**
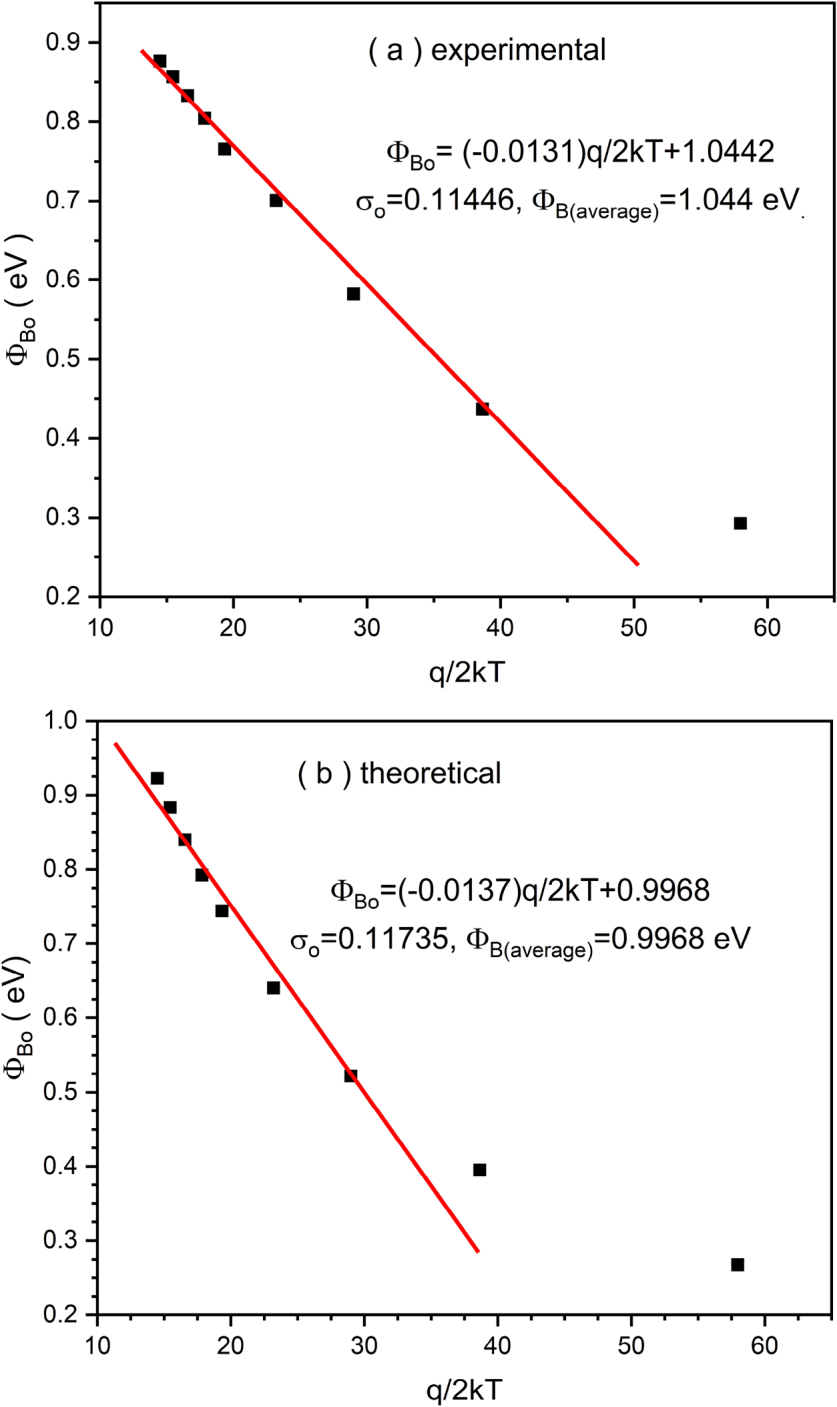
Comparative analysis of experimental and theoretical Schottky barrier height distributions via Gaussian distribution.

From this expression, the standard deviation of the barrier height distribution σ is extracted as 0.11446 eV, and the average barrier height (ϕ_*B*0(average)_) is calculated to be 1.044 eV. The negative slope indicates the presence of spatial inhomogeneities at the metal–semiconductor interface, as expected in real devices. In Figure 6b., the theoretical prediction similarly shows a linear trend

15 $$\phi_{B0}=\left(-0.0137\right)q/2\mathrm{kT}+0.9968$$

Here, the extracted σ is 0.11735 eV, and the corresponding ϕ_*B*0(average)_ is 0.9968 eV. The theoretical model predicts a slightly steeper slope than the experimental result, suggesting a marginally higher degree of barrier inhomogeneity. Additionally, the theoretical average barrier height is lower by approximately 0.047 eV compared to the experimental value. The proximity of both σ values indicates that the Gaussian distribution model provides a reasonably accurate description of the experimental behavior. However, the discrepancy in average barrier height may be attributed to factors such as interface states, local variations in work function, or defects not fully accounted for in the theoretical framework. The comparison confirms that while the theoretical model captures the essential trend of barrier height variation with temperature, slight deviations persist, showing the importance of experimental validation in Schottky contact analysis.

Figure 7a,b illustrate the modified Richardson plots for the experimental and theoretical data, respectively, incorporating barrier height inhomogeneities as prescribed by the Gaussian distribution model. The modified Richardson equation accounts for the standard deviation of the barrier height (σ) and is expressed as

16 $$\ln(\frac{I_{0}}{T^{2}})-\frac{q^{2}\sigma^{2}}{2k^{2}T^{2}}=\ln(A^{*})-\frac{q\phi_{B0\left(\mathrm{average}\right)}}{\mathrm{kT}}$$

From this equation, the experimental average barrier height (ϕ_*B*0(average)_) is determined to be 1.014 eV, and the modified Richardson constant A* is calculated as 113.80 A/cm^–2^K^–2^. The linearity of the data confirms the validity of the Gaussian distribution model for the description of the spatial inhomogeneities in the Schottky barrier. The theoretical average barrier height case is slightly lower, at 1.011 eV, while the theoretical Richardson constant A* is slightly higher, at 118.71 A/cm^–2^K^–2^. The slope and intercept closely match the experimental trend, suggesting a good agreement between model predictions and measured data. The proximity of the slopes (−1.0138 and −1.0109) and the average barrier heights (1.014 vs 1.011 eV) between experimental and theoretical plots shows the robustness of the thermionic emission model, when modified to include barrier inhomogeneities.^77^ However, the small difference in intercept values leads to slightly differing Richardson constants, which may result from deviations such as interface states, barrier thinning, or deviations in effective mass not accounted for in the theoretical model. Despite these minor discrepancies, both plots exhibit strong linearity, validating the assumption of GD barrier heights and confirming the temperature-dependence of the Schottky characteristics.

**Figure 7:**
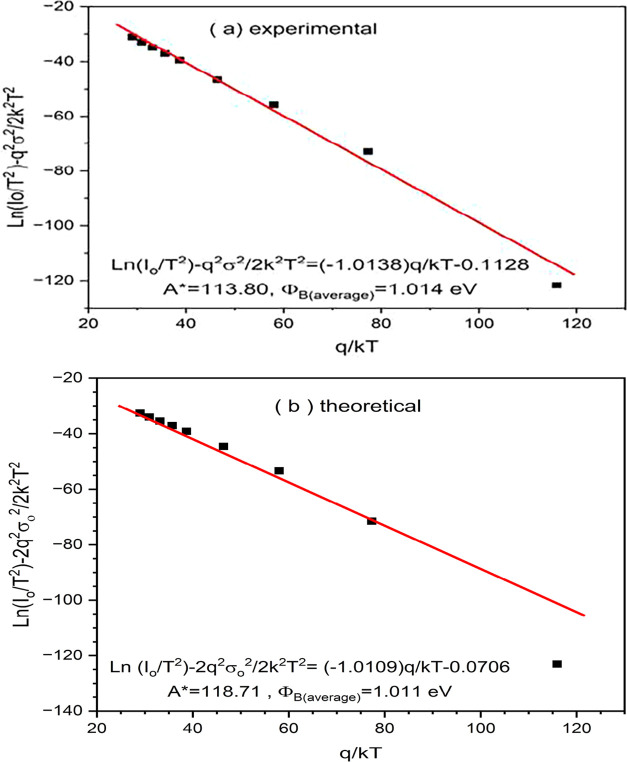
(a) Experimental, (b) theoretical analysis comparative evaluation of modified Richardson plots.

Table 2 presents the temperature-dependent variation of the reverse saturation current (*I*_0_), ideality factor (*n*), and zero-bias barrier height (ϕ_*B*0_) for both experimental and theoretical analyses. Across the entire temperature range (100–400 K), the reverse saturation current increases exponentially with temperature in both data sets, consistent with the thermionic emission model. However, the theoretical *I*_0_ values are significantly higher than the experimental ones, particularly at lower temperatures. For example, at 100 K, the experimental *I*_0_ is 1.61 × 10^–11^ A, whereas the theoretical value is 2.81 × 10^–10^ A. This discrepancy suggests that the theoretical model, which assumes ideal conditions, does not fully account for recombination, interface states, or series resistance effects that are present in real devices.^78^

**Table 2:** Comparative Analysis of Experimental and Theoretical Parameters Derived from *I*–*V* Characteristics

	experimental			theoretical		
*T* (K)	*I*_0_ (A)	*n*	ϕ_*B*0_ (eV)	*I*_0_ (A)	*n*	ϕ_*B*0_ (eV)
100	1.61 × 10^–11^	4.877	0.293	2.81 × 10^–10^	3.299	0.268
150	4.19 × 10^–11^	3.4767	0.437	1.08 × 10^–9^	2.255	0.395
200	7.69 × 10^–11^	2.441	0.582	2.52 × 10^–9^	1.733	0.522
250	4.21 × 10^–10^	1.767	0.701	6.99 × 10^–9^	1.420	0.640
300	1.11 × 10^–8^	1.357	0.766	2.54 × 10^–8^	1.212	0.744
325	3.22 × 10^–8^	1.289	0.804	4.91 × 10^–8^	1.134	0.792
350	1.12 × 10^–7^	1.269	0.833	8.96 × 10^–8^	1.063	0.840
375	3.9 × 10^–7^	1.260	0.857	1.69 × 10^–7^	1.016	0.884
400	1.29 × 10^–6^	1.250	0.877	3.41 × 10^–7^	0.997	0.923

The ideality factor shows a decreasing trend with increasing temperature in both cases, which is characteristic of Schottky diodes. At lower temperatures, high *n* values are observed due to enhanced contributions from recombination and tunneling mechanisms. The experimental ideality factor decreases from 4.88 at 100 K to 1.25 at 400 K, while the theoretical values range from 3.30 to 0.997 over the same temperature interval. The fact that experimental *n* remains above unity even at higher temperatures indicates the persistence of nonideal effects such as barrier inhomogeneities or interface traps, whereas the theoretical model converges to nearly ideal behavior.^79,80^ The zero-bias barrier height (ϕ_*B*0_) increases monotonically with temperature in both data sets, supporting the presence of a Gaussian distribution of barrier heights. At 100 K, the experimental ϕ_*B*0_ is 0.293 eV, increasing to 0.877 eV at 400 K, while the theoretical values range from 0.268 to 0.923 eV over the same temperature span. This increase is attributed to the fact that, at low temperatures, current transport is dominated by regions with lower local barrier heights, whereas at higher temperatures, carriers can surmount higher barriers, leading to an increase in the apparent barrier height. The close agreement between experimental and theoretical ϕ_*B*0_ values, particularly at higher temperatures, demonstrates that the theoretical model successfully captures the essential physics of the barrier inhomogeneity mechanism. The theoretical results show good qualitative agreement with experimental findings, especially in terms of the temperature dependence of ϕ_*B*0_ and *n*. Nonetheless, quantitative differences in *I*_0_ and *n* (especially at low temperatures) shows the limitations of ideal thermionic emission models. These observations confirm the necessity of considering barrier height inhomogeneities and nonideal transport mechanisms for accurate characterization of Schottky diodes.

The systematic discrepancies between the experimental and theoretical parameters, particularly pronounced at low temperatures, can be attributed to the dominance of physical mechanisms not fully captured by the standard thermionic emission model, even when modified for barrier inhomogeneity. For the ideality factor (*n*), the significantly higher experimental values (e.g., 4.88 vs 3.30 at 100 K) are a direct signature of nonideal transport pathways becoming dominant. At cryogenic temperatures, the contribution of trap-assisted tunneling (TAT) and multistep recombination via interface states increases substantially, as the thermal energy required for pure thermionic emission is insufficient.^81–83^ These mechanisms effectively shunt the barrier, leading to excess current and a higher apparent ideality factor. The model, while accounting for a distribution of barrier heights, does not incorporate the explicit physics of defect-mediated tunneling, leading to an underestimation of “*n*”.

The deviation in the saturation current (*I*_0_) originates from two principal limitations in the theoretical framework. First, the model assumes an idealized carrier mobility that does not adequately reflect low-temperature transport phenomena.^84,85^ At reduced temperatures, impurity scattering, carrier freeze-out, and suppressed phonon interactions significantly degrade mobility, yet these effects are not explicitly incorporated into the simulation. As a result, the calculated *I*_0_ remains artificially elevated under cryogenic conditions. Second, the adoption of a constant Richardson constant across all temperatures introduces systematic inaccuracies. In heterostructures such as AlN/n-Si, both the effective mass and the density of states exhibit temperature-dependent behavior due to band structure evolution and interfacial effects.^86,87^ Since the Richardson constant is intrinsically linked to these parameters, treating it as invariant fails to capture the actual thermionic emission dynamics, leading to persistent discrepancies between theoretical and experimental *I*_0_ values.

Regarding the zero-bias barrier height (ϕ_*B*0_), the consistent offset points to an incomplete description of the interface electrostatics. The model’s reliance on a single, temperature-independent standard deviation (σ) for the Gaussian distribution of barrier heights is a simplification. In reality, the distribution itself may contract with decreasing temperature due to the reduced influence of localized phonon modes and interface dipoles.^88–90^ Furthermore, the extraction of ϕ_*B*0_ from experimental *I–V* curves is influenced by the very same TAT and recombination currents that inflate the ideality factor, leading to an overestimation of the apparent barrier height compared to the theoretical value, which represents a purely thermionic average.

In summary, the theoretical model provides an excellent framework for understanding the thermionic contribution to transport and the role of barrier inhomogeneity. The quantitative mismatches, however, serve as a quantitative measure of the increasing contribution of nonthermionic, defect-driven conduction mechanisms at low temperatures, showing the complex nature of charge transport in real-world semiconductor heterojunctions.

## 4 Conclusions

In this investigation, both experimental measurements and theoretical modeling were conducted to examine the temperature-dependent electrical behavior of a Au/Ti/AlN/n-Si Schottky diode. Fundamental parameters, including the reverse saturation current, ideality factor, and zero-bias barrier height were extracted across a broad temperature range (100–400 K) and analyzed within the context of thermionic emission theory, accounting for Gaussian-distributed barrier height inhomogeneities. The experimental findings demonstrated a pronounced temperature dependence of all extracted parameters. Notably, the ϕ_*B*0_ exhibited a steady increase with rising temperature, indicative of an inhomogeneous Schottky interface and consistent with a distribution of local barrier heights at the metal–semiconductor junction. This trend was well captured by the theoretical model, which yielded closely matching ϕ_*B*0_ values at higher temperatures. Modified Richardson plots were employed to account for the inhomogeneous nature of the barrier, and the extracted average barrier heights and Richardson constants supported the applicability of the Gaussian distribution model. While the theoretical results closely matched the experimental trends, discrepancies at low temperatures show the limitations of idealized models and the influence of interface states and structural imperfections in devices. The comparison between experimental and theoretical data demonstrates that modeling barrier height inhomogeneities is essential for accurately describing Schottky diode behavior. The findings contribute to a deeper understanding of transport mechanisms in metal–semiconductor contacts and provide a foundation for improving the performance and reliability of Schottky-based electronic devices.
