# Correction to “Investigation of Electrical Behavior of Au/Ti/AlN/Si Schottky Diode via Gaussian Distribution Barrier Modeling”

**DOI:** 10.1021/acsomega.6c10765

**Published:** 2026-09-30

**Authors:** Abdullah Karaca, Dilber Esra Yıldız, Adem Tataroğlu

We are issuing a correction to the author affiliation of the original manuscript (DOI: [10.1021/acsomega.5c07643]). The second author affiliation of Prof. Dr. Dilber Esra Yıldız was removed.

- **Requested Correction Details:**

**Author:** Prof. Dr. Dilber Esra Yıldız

**Second Affiliation to Be Removed:**

“Department of Chemistry, Faculty of Arts and Sciences, Middle East Technical University, 06800 Ankara, Turkey” was deleted.

We apologize for the oversight.

